# Obesogenic environments: a systematic review of the association between the physical environment and adult weight status, the SPOTLIGHT project

**DOI:** 10.1186/1471-2458-14-233

**Published:** 2014-03-06

**Authors:** Joreintje D Mackenbach, Harry Rutter, Sofie Compernolle, Ketevan Glonti, Jean-Michel Oppert, Helene Charreire, Ilse De Bourdeaudhuij, Johannes Brug, Giel Nijpels, Jeroen Lakerveld

**Affiliations:** 1 The EMGO Institute for Health and Care Research, Department of General Practice and Elderly Care Medicine, VU University Medical Center, Amsterdam, The Netherlands; 2 European Centre on Health of Societies in Transition, London School of Hygiene and Tropical Medicine, London, UK; 3 Department of Movement and Sport Sciences, Faculty of Medicine and Health Sciences, Ghent University, Ghent, Belgium; 4 Université Paris 13, Sorbonne Paris Cité - UREN (Unité de Recherche en Epidémiologie Nutritionnelle), U557 Inserm; U1125 Inra; Cnam, Centre for Research on Human Nutrition Ile-de-France (CRNH IdF), Bobigny, France; 5 Université Pierre et Marie Curie-Paris 6, Dept of Nutrition Pitié-Salpêtrière Hospital (AP-HP), (CRNH IdF), Institute of Cardiometabolism and Nutrition (ICAN), Paris, France; 6 Research Unit on Nutritional Epidemiology INSERM U557, Paris 13, Human Nutrition Research Center of Ile de France and Paris Est University, Lab-Urba Urban Institut of Paris, UPEC, Créteil, France; 7 The EMGO Institute for Health and Care Research, Department of Epidemiology and Biostatistics, VU University Medical Center, Amsterdam, The Netherlands

**Keywords:** Review, Physical environment, Overweight, Obesity, Adults, Quality assessment

## Abstract

**Background:**

Understanding which physical environmental factors affect adult obesity, and how best to influence them, is important for public health and urban planning. Previous attempts to summarise the literature have not systematically assessed the methodological quality of included studies, or accounted for environmental differences between continents or the ways in which environmental characteristics were measured.

**Methods:**

We have conducted an updated review of the scientific literature on associations of physical environmental factors with adult weight status, stratified by continent and mode of measurement, accompanied by a detailed risk-of-bias assessment. Five databases were systematically searched for studies published between 1995 and 2013.

**Results:**

Two factors, urban sprawl and land use mix, were found consistently associated with weight status, although only in North America.

**Conclusions:**

With the exception of urban sprawl and land use mix in the US the results of the current review confirm that the available research does not allow robust identification of ways in which that physical environment influences adult weight status, even after taking into account methodological quality.

## Background

Obesity prevention is a global public health priority as a result of the worldwide increase in obesity prevalence [1] and its associated chronic diseases [2]. Although genetic factors may underlie the propensity of individuals to become obese [3], the pace at which obesity prevalence has grown at population level during recent decades points to social and environmental causes [4,5]. An individual’s body mass index (BMI) is mainly determined by energy intake (eating) and energy expenditure (physical activity/sedentary behaviour). These energy balance related behaviours (EBRBs) are influenced by a range of determinants [6]. One important category of determinants is the opportunities for calorie intake and calorie expenditure or a lack thereof in the physical environment. For example, dietary behaviour may be influenced by access to different foods through various types of outlets and services. Similarly, physical activity levels may be influenced by access to recreational or sports facilities, green spaces or parks, as well as transport infrastructure and land use. Certain environments may be more ‘obesogenic’ than others, such that they are more likely to promote weight gain and obesity in individuals or populations [5], but it remains a challenge to identify the physical environmental factors with the greatest impacts on (the development of) overweight and obesity.

Following a steady increase in studies relating characteristics of the physical environment to overweight or obesity in the last decade, a number of reviews was published between 2005 and 2012 [7-12], showing mixed results. Among a large range of factors that have been examined, only two environmental correlates appeared to be consistently associated with weight status: indicators of urban sprawl (often based on population density, and positively associated with obesity) and measures of land use mix (negatively associated with obesity) [12,13]. However, there are numerous potential correlates of obesity from the physical environment and it is plausible that many other environmental factors such as access to recreation areas, proximity to fast food outlets or the presence of walking and cycling infrastructure might influence weight status through their links to food and physical activity behavior [14-17]. It may be that the heterogeneity in methods and measures used, or differences in contexts or location, has led to this lack of consistent results.

A recent review of reviews identified a number of gaps and areas for improvement that could explain some of the inconsistencies in the findings about obesogenic environments [12]. The authors evaluated the quality of previous literature reviews on built environment, physical activity and obesity based on: 1) the age and other demographic characteristics of the population, 2) the time frame for the literature search, 3) the total number of articles included, 4) data sources, 5) whether the methodological quality of the primary studies was assessed, 6) whether the measurement mode of the characteristics of the physical environment was reported, 7) whether the outcome was defined, and 8) whether the measurement mode of the outcome was reported. The methodological quality of the primary studies had been assessed in very few of the reviews identified [12]. Although quality assessment tools are rarely used in the evaluation of observational studies [18], assessing the methodological quality of any included studies is an important element of systematic literature reviews.

In addition, previous reviews have generally not distinguished between objective and perceived measures of the environment. Aspects of the physical environment are considered to be measured objectively when assessed through street audits, virtual audits (using remote imaging data) or on the basis of Geographic Information Systems [19,20]. The agreement between these objectively measured aspects of physical environments and perceptions of these environments (as measured with interviews or questionnaires) is generally considered to be moderate or low [21,22].

Consequently, we aim to provide an updated review of the literature on physical environmental correlates of adult weight status. We gave a detailed overview of the characteristics of the primary studies and assessed the methodological quality of the included articles. This quality assessment allowed comparison of results of studies that are methodologically strong with those that are methodologically weaker. On the basis that environments are likely to be very different in high income countries from those in middle or low income countries, in this review we have focused on studies that were conducted in high income countries. In addition, we stratified the included studies by mode of measurement and continent of origin.

## Methods

Original studies that examined associations between physical environmental characteristics and adult weight status were reviewed. The physical environment was defined as all built environmental and transport related factors [5]. A literature search, using five electronic databases (PubMed, EMBASE, Web of Science, Cochrane Library and PsychInfo), was conducted in May 2013. Studies relating a physical environmental factor to BMI, overweight or obesity in adults were considered if published between January 1995 and May 2013 in Dutch, English, French or German language. A full description of search terms and search strategy is provided in Additional file 1. Articles were included if weight status in adults was one of the main outcomes. Furthermore, we included articles that focused on macro environmental correlates (i.e. environmental factors measured at the neighbourhood, province or national level) of weight status. Articles of studies were excluded if they:

● focused primarily on socioeconomic characteristics or the social environment of a geographic area

● assessed a physical environmental factor only as a potential confounder

● had a very specific target population that would lead to non-generalizable results (e.g. senior citizens, pregnant women, particular ethnic groups in specific locations, etc.)

● were conducted in low or middle income countries or regions

● did not present original research (e.g. reviews, case reports, editorials, commentaries, discussions or letters)

### Data extraction

The following information was extracted from included studies:

● country (and continent) where the data were collected

● study design

● number of participants

● main environmental determinants

● whether characteristics of the physical environment were measured objectively or subjectively

● whether weight status was measured or self-reported

● whether the reported associations were or were not in agreement with the hypothesis stated in the article, or whether no association was found

● how the scale of the geographical study area was defined.

The first table presents studies from North America, Australasia, and Europe. Study design was divided into cross-sectional or longitudinal. Physical environmental determinants were categorized into four domains: 1) Physical activity environment, referring to physical activity opportunities, 2) Food environment, referring to food purchasing opportunities, 3) transport opportunities and 4) other (e.g. population density). The definition of the scale of geographical areas was divided into studies that used administrative area limits (such as census tracts or counties), studies that used buffers (for example a 1 km circular or network buffer around the home) or studies that used a non-standard definition of neighbourhood (for example self-perceived neighbourhood limits).

### Assessment of the methodological quality

For quality assessment, we adapted the Quality Assessment Tool for Quantitative Studies (as developed by the Effective Public Health Practice Project) [23], based on recommendations from a number of authors [24-27]. This contains 19 items in eight key domains for assessment of study quality: 1) study design, 2) blinding, 3) representativeness in the sense of selection bias, 4) representativeness in the sense of withdrawals/drop outs, 5) confounders, 6) data collection, 7) data analysis and 8) reporting and is suitable for assessing observational, as well as experimental studies. Studies can have between six and eight component ratings. An overall rating for each study was determined based on the component ratings, ranging from 1 (low risk-of bias; high methodological quality) to 3 (high risk-of-bias; low methodological quality). For example, if eight ratings were given, *strong* was attributed to those with no weak ratings and at least five strong ratings, *moderate* was given to those with one weak rating or fewer than five strong ratings and *weak* was attributed to those with two or more weak ratings. The quality assessment tool we used is presented in full in Additional file 2.

All included studies were independently assessed for methodological quality by two assessors (JDM and KG). The ratings for each of the eight domains, as well as the total rating, were compared between the two assessors. Consensus was reached on a final rating for each included article.

## Results

After duplicates were removed, 5,642 articles were screened on title and abstract by the first author. Subsequently, a random sample of 500 titles and abstracts was also screened by the second author; 212 full articles were read by the first and second author. Of these articles, 92 were included in the review (see Flow chart in Additional file 3). Characteristics of the studies are shown on Table 1.

**Table 1:** Characteristics of included studies

**First author**	**Year**	**Country**	**Design†**	**N**	**Domain**^**‡**^					**Main correlates**^**Ω**^	**O/P**^**¥**^**measure of PE**	**M/S**^**¥**^**weight status**	**Association**^**‡**^			**Definition area**^**§**^
					***PA envir***	***Food envir***	***Trans-port***	***Urban Form***	***Other***				***expec-ted***	***null***	***unex-pected***	
Northern America																
Ahern [28]	2011	USA	CS	3,128		x				Restaurants and grocery stores	O	S	x			County
Black [29]	2009	USA	LG	48,506	x	x			x	Stores, facilities, crime	O	S	x			Census nbh
Black [30]	2010	USA	CS	9,916	x	x				Food avail, opportunities & barriers to PA	O	S	x		x	Census nbh
Block [31]	2011	USA	LG	3,113		x				Proximity to food establishments	O	M	x	x		Census nbh
Bodea [32]	2009	USA	CS	6,893	x			x		Street connectivity, residential density	O	S	x			1 km buffer
Bodor [33]	2010	USA	CS	3,925	x					Food environment	O	S	x	x		2 km buffer
Brown [34]	2009	USA	CS	5,000	x		x	x		Walkable land use, destinations	O	S	x	x	x	Census block group
Brown [35]	2013	USA	CS	3.528	x					Walkability, bikeability	O	M	x			Census block group
Casagrande [36]	2011	USA	CS	3,493	x					Walkability	O	M		x		Census nbh
Chen [37]	2012	USA	CS	3,550		x				Food outlets	O	S	x	x		0.5 mile buffer
Doyle [38]	2006	USA	CS	9,252	x				x	Walkability, crime	O	M	x			County
Drewnowski [39]	2012	USA	CS	1,682		x				Proximity and price of supermarkets	O	S			x	1 mile distance to supermarket
Eid [40]	2008	USA	CS	5,500				x		Urban sprawl	O	S		x		2 mile radius disc
Ewing [41]	2003	USA	CS	206,992				x		Urban sprawl	O	S	x			County
Frank [42]	2004	USA	CS	10,878	x			x		Land use mix	O	S	x			Block group/square kilometer
Frank [43]	2008	USA	CS	13,065				x		Residential density,, street connectivity, land use mix	O	S	x			1 km distance
Frank [44]	2007	USA	CS	2,056	x					Walkability	O	S	x			Road polygons traveling 1 km from house
Frank [45]	2007	USA	CS	1,228	x					Walkability	O	S	x			1 km network buffer
Frank [46]	2009	USA	CS			x				Food outlet visits, walkability	O	S	x	x		1 km
Gibson [47]	2011	USA	LG	8,100		x				Food environment	O	S	x			ZIP code area
Gregson [48]	2011	USA	CS	14,205		x		x		Sprawl and restaurant types	O	S	x		x	County
Hattori [49]	2013	USA	CS	97,678		x				nbh food outlets	O	S		x		1 mile Euclidian distance
Hoehner [50]	2011	USA	CS	17,000	x					Walkability	O	M	x			Block group
Hutchinson [51]	2012	USA	CS	1,243		x				Availability of healthy & unhealthy foods	O	S	x	x		Census tract
Inagami [52]	2009	USA	CS	2,156		x				Fast food & restaurant concentration	O	S	x			Census tract
James [53]	2012	USA	CS	68,000				x		County sprawl index	O	S	x			County
Jeffery [54]	2006	USA	CS	1,033		x				Proximity of (fastfood) restaurants	O	S		x		0.5 miles, 1 miles and 2 miles from home and work adresses
Jilcott [55]	2010	USA	CS	9,800		x				Food retail gap	O	S	x			County
Keegan [56]	2012	USA	CS	133,000				x	x	Population & housing density, commuting characteristics	O	S		x		Census nbh
Lesser [57]	2013	USA	CS	2,589		x				Outdoor food advertising	O	S	x			Census tract
Lopez [58]	2004	USA	CS	104,084				x		Urban sprawl	O	S	x			Tracts of approx 4000 people
Lopez [59]	2007	USA	CS	15,358	x	x		x	x	Various	O	S	x	x		ZCTA
Lovasi [60]	2009	USA	CS	13,102	x					Walkability	O	M	x			1 km buffer
Lovasi [61]	2012	USA	CS	13,102	x		x		x	Walkability, aesthetics, safety	O	M	x	x	x	1 km buffer
McDonald [62]	2012	USA	CS	690	x					Walkability	O	M		x		Census block group
Mehta [63]	2008	USA	CS	714,054		x				Restaurant mix	O	S	x			County
Mobley [64]	2006	USA	CS	2,692	x	x		x	x	Fitness facilities, food establish-ments, crime	O		x	x		ZIP code areas
Morland [65]	2006	USA	CS	10,763		x				Food stores	O		x			Census tract
Oka [66]	2012	USA	CS	5,485	x	x	x			Various	O	M		x		Census tract
Plantinga [67]	2007	USA	LG	4,700				x		Sprawl	O	S	x			County
Plantinga [68]	2007	USA	CS	3,607				x		Sprawl	O	S		x		County
Rundle [69]	2007	USA	CS	13,102	x		x	x		Land use, bus stop density, pop density, intersection density	O	M	x	x		Census tract area
Rundle [70]	2008	USA	CS	13,102	x	x				Density of BMI-healthy food outlets and walkability	O	M	x	x		805 meter network buffer
Rundle [71]	2009	USA	CS	13,102	x	x				Food environment and walkability	O	M	x	x		Half-mile radius circular buffers
Rundle [72]	2013	USA	CS	13,102	x					Park characteristics	O	M	x	x		Half-mile radius circular buffers
Sallis [73]	2009	USA	CS	2,199	x					Walkability	O	S	x			Block groups
Samimi [74]	2009	USA	CS	300,000			x	x		Transport, land use, built environment	O	S	x			Census tract
Scott [75]	2009	USA	CS	1,750	x			x	x	Safety, des- tina- tions, social fac- tors	O	S	x	x		Urban census tract
Smith [76]	2008	USA	CS	453,927	x					Walkability	O	S	x	x		Census block group
Smith [77]	2011	USA	CS	100,000	x					Walkability	O	S	x	x		Census block group
Wang [78]	2007	USA	CS	7,595		x				Proximity & density of fastfood restaurants & food retail	O	S	x	x		Combination of census tracks and block groups
West [79]	2012	USA	CS	99,534	x					Park(land) area	O	S	x			metropolitan statistical area
Yamada [80]	2011	USA	CS	4,960				x		mixed land use	O	S	x			Block group, tract, 1 km buffer
Zhao [81]	2010	USA	LG					x		Urban sprawl	O	S	x			Census metropolitan areas/county level
Zick [82]	2009	USA	CS	453,927	x	x				Food and PA opportunities	O	S	x	x		Block groups
Zick [83]	2013	USA	CS	35,685	x					Walkability	O	S	x			Census block
Bai [84]	2013	USA	CS	893	x					Park quality	P	S	x	x		Census block within 0.5 miles of park
Catlin [85]	2003	USA	CS	2,821	x	x				Community perceptions, community infrastructure, PA facilities	P	S	x	x		Community
Mujahid [86]	2008	USA	CS	2,865	x	x			x	Neighborhood conditions	P	M	x	x		Census tract
Powell-Wiley [87]	2013	USA	CS	5,907	x	x			x	nbh violence, physical environment, social cohesion	P	M	x	x		Neighborhood
Wilson [88]	2007	USA	CS	1,111	x				x	opportunities for PA and pleasant neighbourhoods	P	S	x	x		Neighborhood and community
Tilt [89]	2007	USA	CS	529	x					greenness, accessibility	O&P	S	x	x		0.4 mile distance
Boehmer [90]	2007	USA	CS	1,032	x		x	x	x	Facilities land use, transportation, aesthetics	O&P	S	x	x	x	400 m radius
Rutt [91]	2005	USA	CS	996	x			x	x	Various	O&P	S		x		1/4 mile radius and 2.5 mile radius
Pendola [92]	2007	USA	CS	670				x	x	Population density, sense of community	O&P	S		x		Census tract
Joshu [93]	2008	USA	CS	1,818				x		Sprawl, urbanization	O&P	S	x			FIPS code
Berry [94]	2010	Canada	CS & LG	572 and 1,164	x					Ease of walking and proximity to outdoor recreation	O	S		x		Census nbh
Spence [95]	2009	Canada	CS	2,900		x				RFEI (retail food environment index)	O	S	x	x		800 m and 1600 m
Pouliou [96]	2010	Canada	CS	115,548	x	x		x		Built environment	O	S	x			Subprovincial scale
Prince [97]	2012	Canada	CS	6,564	x	x			x	Recreation, social, food environment	O	S	x	x		Neighborhoods based on natural barriers?
Prince [98]	2011	Canada	CS	5,025	x	x			x	Recreation, social, food environment	O	S	x	x		Neighborhoods based on natural barriers?
Ross [99]	2007	Canada	CS	33,000				x	x	Dwelling density, sprawl, immigration	O	S	x	x		Census tract area
Kestens [100]	2012	Canada	CS	5,578		x				Food environment	O	S	x	x		Census nbh
Berry [101]	2010	Canada	CS & LG	500	x					Perceptions of neighbourhood	O&P	S		x		Census nbh
																
Australasia																
Garden [102]	2008	Australia	CS	7,290				x		Urban sprawl	O	S	x			Local government area
Pearce [103]	2009	New Zealand	CS	12,529		x				distance to fastfood outlet	O	M		x	x	meshblock neighbourhoods
Richardson [104]	2013	New Zealand	CS	8,157	x					Urban green space	O	M		x		Census area unit
Christian [105]	2011	Australia	CS	1,151	x	x				Built & social environment	O&P	S	x	x		1.6 km road network service area
Gebel [106]	2011	Australia	LG	1,027	x			x		Walkability, dwelling density, land use mix	O&P	S	x	x		Census collector district
Giles-Corti [107]	2003	Australia	CS	1,803	x					Physical environment	O&P	S	x			Collector districts
																
Europe																
Ball [108]	2012	UK	CS	1,062	x					Street connectivity	O	S			x	Datazone
Van Dyck [109]	2010	Belgium	CS	1,200	x					Walkability	O	S		x		Statistical sectors
Santana [110]	2009	Portugal	CS	7,669	x	x		x	x	Environmental disadvantages & opportunities	O	S	x	x		Neighbourhood?
Ellaway [111]	2005	Europe	CS	6,919	x				x	Graffiti, greenery	O	S	x			immediate residential environment
Macdonald [112]	2011	UK	CS	991		x				Distance to food stores	O	S		x	x	500 and 1000 meter
Leal [113]	2012	France	CS	7,230				x	x	Sociodemographic factors, physical factors, service-related, social-interactional environment	O	M	x	x		500 m radius/TRIRIS geographic unit
Burgoine [114]	2011	UK	CS	893		x		x		Residential density, street connectivity, land use mix	O	S	x	x		LSOA/MSOA
Coombes [115]	2010	UK	CS	6,821	x					Green space	O	S	x			Not applicable (distance)/800 meter
Cummins [116]	2012	UK	CS	79,136	x					Percentage greenspace	O	S		x	x	MSOA
Poortinga [117]	2006	UK	CS	14,836				x	x	Friendliness, trust, social capital, access	P	M	x	x		Postcode sectors
Toftager [118]	2011	Denmark	CS	21,832	x					Distance to green space	P	S	x			Not applicable (distance)
Nielsen [119]	2007	Denmark	CS	2,000	x					Perceptions of distance to garden/green area	P	S	x			Not applicable (distance)

† CS = cross-sectional, LG = longitudinal.

‡ x = association was statistically significant in this direction.

¥ Measures of the Physical Environment (PE): O = objective, P = perceived. Measures of weight status: M = objectively measured, S = self-reported.

Ω PA = physical activity, nbh = neighbourhood.

§ nbh = neighbourhood, km = kilometer, m = meter, LSOA/MSOA = lower/middle statistical output area, ZCTA = Zip Code Tabulation Area.

A large majority of studies (74) was conducted in North America (USA: 66, Canada: 8), 12 were conducted in Europe (half in the UK) and six were conducted in Australasia. Half the studies (45) were published from 2010 onwards, with seven in 2013. Before 2010, 47 studies were published. Most studies (75) used exclusively objective measures of the physical environment, while 17 studies used perceived measures to link physical environmental characteristics to weight status. Of these 17 studies, nine studies examined both the objective and the perceived environment.

There was great heterogeneity across studies in the use and definition of physical environmental factors. Fifty-three studies investigated the association between an environmental factor that was presumed to affect obesity through physical activity (such as parks or sports facilities), thirty-six studies assessed the association of the food related environment (such as the density of fast-food restaurants) and six studies assessed the transport-related environment (such as proximity to public transport amenities). Thirty-one studies assessed urban form characteristics such as street connectivity, urban sprawl and land use mix, and twenty studies investigated other types of environmental factors such as graffiti or crime. Fifteen studies assessed associations between both food related and physical activity related environments and obesity. It goes beyond the scope of this review to present all the different metrics used, but an overview of the different metrics and associated operationalization of physical environmental factors used in these kind of studies has been provided elsewhere [9]. As an example of the heterogeneity between studies, while fifty-three studies investigated a physical activity related environmental factor, none of the seven [79,89,104,111,115,116,119] studies examining green space used the same definition of this metric. Tilt et al. calculated greenness with NDVI (normalized difference vegetation index; the amount of photosynthetically active light as measured with infrared light) [89], while West et al. defined green space as all publicly owned and operated green spaces [79]. Toftager et al. included beaches, seas, forests and lakes but no agricultural fields [118], while Cummins et al. included agricultural land but excluded domestic gardens [116]. The remaining three studies also used different definitions of green space.

There was no consistent pattern of associations between physical environmental factors and weight status. For example, nineteen studies assessed the association between walkability (often a combination of three factors: intersection density, land use mix and population density) and overweight or obesity – one study from Europe and 18 studies from North America. The European study did not find an association between walkability and obesity, although there was an association between walkability and several domains of physical activity [109]. Of the other 18 studies, eight found associations that were in line with the hypothesis (i.e. higher walkable areas are associated with lower BMI or overweight prevalence). Three studies did not find statistically significant associations and five studies found inconsistent results. Inconsistencies arose from results that indicated that associations were only present in men versus women, only present in disadvantaged areas versus higher SES neighbourhoods, or only present for BMI versus overweight/obesity as outcome.

Only eight studies used longitudinal data. The follow-up time ranged from four to 25 years. Sixty-six studies defined the geographical scale of study (‘neighbourhood’ or ‘environment’) based on administrative boundaries (for example: county or census tract). The remaining studies used ‘buffers’ (network or Euclidian) with varying radius, with the exception of one study that used limits of activity-space. Of the 93 included studies, 36 presented results that broadly corresponded with the hypothesis in the study (i.e. the results were according to what was expected). Fourteen studies reported that they did not find statistically significant results and 5 studies reported unexpected results, i.e. opposite to the hypothesised direction. Another 38 studies reported on a mixture of expected, unexpected and/or non-significant results.

### Intercontinental differences

Two environmental measures were relatively consistently and statistically significantly associated with overweight status or BMI; urban sprawl and land use mix. Urban sprawl was studied in twelve studies (none of the European studies, 1 Australian study and 11 North American studies): eight reported on significant associations in the expected direction (more urban sprawl was related to more obesity), and four reported no association. Land use mix (separately from walkability) was examined in five North American studies, and was significantly associated with obesity in all of these studies (less land use mix was related to more obesity). One study from the UK showed that land use mix was not significantly associated with overweight or obesity [114]. However, because of the dominance of North American studies, it was not possible to differentiate between studies from European, Australasian or North American origin.

In Europe, five [111,115,116,118,119] out of twelve studies investigated the role of green space in the risk for overweight or obesity. Although Nielsen & Hansen, Toftager and Ellaway et al. found results supporting the hypothesis that green space is associated with lower BMI [111,118,119], the results of Cummins & Fagg and Coombes et al. did not support this hypothesis [115,116]. Other European studies did not provide clear evidence about any physical environmental factors associated with overweight or obesity.

It was not possible to conclude which physical environmental factors were specific to Australasia with regards to the relation between characteristics of the physical environment and obesity. The two studies from New Zealand examined fast-food outlets [103] and green space [104] (no significant associations) and the four studies from Australia examined several physical environmental factors such as type of street, spatial access to natural facilities, graffiti and street connectivity.

### Objective versus perceived measures

Six studies used only perceived measures of the environment (for example; perceived distance to green space or perceptions of access to amenities), but the range of factors studied was broad: in total, over 20 different factors were examined in these six studies. Nine studies assessed both perceived and objectively measured environment, but most studies did not assess the same factors objectively as subjectively. Tilt et al. and Boehmer et al. were the only authors who were able to compare the objective and subjective measures. Tilt et al. concluded that only objectively measured factors (accessibility and greenness) were associated with BMI [89], while Boehmer et al. concluded that perceived as well as objective measures of land use and aesthetics were the most robust correlates of obesity, compared to a range of other factors [90]. Gebel et al. used an alternative approach by showing that a mismatch between objectively measured and perceived walkability measures was associated with weight gain: those who perceived a highly walkable area as being of low walkability showed a larger increase in BMI than those with concordant perceptions [106].

### Methodological quality assessment

Overall, for 29 articles the methodological quality was rated as strong, for 53 articles as moderate and for 8 as weak (full details on the quality assessment are provided in Additional file 4). Eleven articles were rated as weak regarding representativeness, and 45 and 31 were rated as strong and moderate respectively regarding representativeness (five articles did not receive a rating because they referred to a design article but did not provide any other information [35,72,82,100,104]). Forty-six articles scored weak on data collection as they did not provide information about the validity or reliability of their measures, twenty-seven scored moderate and nineteen articles scored strong. In terms of confounding five papers were rated as moderate and one as weak as these studies did not adjust for income. All studies except one scored strong on ‘analysis’. Seven studies scored moderate on reporting; all other studies were rated as strong on this issue. There appeared to be no association between the overall methodological quality of the reviewed article and the likelihood of reporting associations that were in line with the authors’ hypotheses. Of the 11 studies that received an overall weak score, three studies solely reported results that were in concordance with the hypothesis. Of the moderate (53) and strong (30) studies, 19 and 10 reported results that were in concordance with the hypothesis, respectively.

## Discussion

We systematically reviewed the published scientific literature on associations between physical environmental factors and weight status in adults. The results showed a great heterogeneity in findings. In line with previous results [4,7,9,10,12], two environmental variables appeared to be more consistently associated with overweight or obesity than other factors: ‘urban sprawl’ and ‘land use mix’. Of note, these two factors have been widely studied in North America, but not in Europe or Australasia. For other environmental variables, there was great variation in the metrics used, the number of features studied and the different contexts of the studies. The current evidence base therefore provides inconsistent results about associations between the physical environment and overweight or obesity in adults.

Previous literature reviews have generally shown a positive association between the physical (or built) environment and obesity. For example, Booth et al. concluded that there was ‘strong preliminary evidence of a relationship between built environment features and the prevalence of obesity’. However, the authors only included 9 papers [4]. Papas et al. included 20 studies, and stated that ‘17 found a statistically significant relation between some aspect of the built environment and risk of obesity’ [7]. Feng et al. included 63 studies, and were cautious in drawing their conclusions: ‘While there is strong intuitive appeal to the notion that the built environment must be contributing to the obesity epidemic, existing scientific evidence does not provide consistent or convincing support for this hypothesis’ [9].

We hypothesized that this lack of apparent inconsistent results was due to heterogeneity in the measures and methods used in primary studies. We therefore conducted stratified analyses, grouping homogeneous studies together in order to identify any patterns that might emerge if we reduced heterogeneity. Although some authors stated that there was limited generalizability of North American results to European settings [7,116], findings remained heterogeneous. As there were not enough studies taking into account similar physical environmental factors between North America, Australia and Europe, it was not possible to make intercontinental comparisons. It may well be that in developed societies, the differences between physical environments within one country or city are not large enough to create measurable differences in impact on outcomes.

Stratification by mode of measurement did not reveal consistent differences between studies that used objective measures versus perceptions of the environment. Although a number of tools to measure perceptions of the environment are available (for example: NEWS [120], ALPHA [121], NWS [122], PANES [123]), many authors used a self-developed instrument, making it difficult to compare results between studies. Comparison of studies that used both objective measures and perceptions of the environment was hampered by to the fact that different dimensions are assessed with these measurement modes. While researchers often objectively assessed distance to a specific facility, they tended to ask for general perceptions of distance: for example ‘is this facility present in your neighbourhood’ instead of ‘do you think this facility is present within 2.5 kilometres. It was noteworthy that no studies reporting on perceptions of the environment found associations that were contrary to the author’s prior hypotheses. This might indicate that unexpected associations were not found, that unexpected associations were not reported (or published), or that prior hypotheses were not tightly defined.

Taking into account the methodological quality of the primary studies did not lead to different results from previous reviews. This may, however, be a function of the approach taken by the tool: it is possible that the determining factor is not the quality of the study, but rather the conceptual model it is based on. As Ding and Gebel [12] describe, we should look for more complex conceptual and statistical models, taking into account innovative analyses and distinguishing between objective and subjective measures of the environment. A relatively simple quality assessment may not be capable of discerning the factors that differentiate these more sophisticated approaches.

Although there is a general consensus that the physical environment has an important influence on individuals’ weight status (in environments where there is no food, one cannot eat; in environments where there are no cars, public transport or machines, one cannot avoid being more physically active for transport, daily activities or work), a large body of research has failed robustly to identify direct causal pathways between the physical environment and weight status. We found no evidence that continent, mode of measurement of the physical environmental correlates, or the methodological quality of primary studies affected the consistency of the results. There may, however, be a number of additional explanations for this lack of consistent associations.

An extra set of quality criteria – specific for studies relating environmental factors to health or weight status – could therefore be defined. This could include taking into account interactions of objective measures and perceptions, the effect of mediators and moderators, and the complexity of conceptual models. Crucial mediators obviously include energy balance related behaviours (EBRBs) such as dietary habits and levels of physical activity, and future studies would benefit from including these kinds of intermediate outcomes. While physical activity mediators have been examined in some studies [36,45,117,118], only two of the reviewed studies took into account food related mediators [86,113]. Furthermore, different environmental variables may moderate each other’s influences or may be moderated by individual level determinants. For example, people with high self- efficacy for physical activity or who perceive strong social support or social pressure to be physical active, may be less influenced by physical environments that do not support physical activity [124] than those with lower levels of such factors. A number of studies that have recently explored mediation and moderation between individual level and family and neighbourhood environmental level determinants have been conducted recently, and indeed suggest such relationships in their associations with EBRB and weight status (i.e. [125]. It may also be necessary to more critically assess the methods used for assessing objective and subjective measures of the environment. Even objective measures are only able to capture part of the ‘true’ physical environment, and perceptions of the environment may be heavily influenced by demographic or lifestyle factors.

Then, it may be that the areas in which the included studies were conducted do not provide adequate variety in exposure to result in measurable differences in outcomes. Indeed, many studies were conducted in only a single city or region. It may be valuable to assess physical environmental factors in a wider region (for example; the approach taken by the SPOTLIGHT project [126]). Additionally, the use of administrative units may be ill-suited to examine environmental effects on health. The impact of exposure to environmental variables in a neighbourhood, area or place may differ between individuals [127,128], so previous studies might have misclassified relevant study areas. Furthermore, it would advance the field if more emphasis were placed on the difference between causation and correlation. Longitudinal observational studies and natural experiments have the advantage of allowing for temporal associations, while accounting for residential self-selection (endogeneity) [73] may also be possible in cross-sectional study designs.

Future researchers should consider the complexity of the relations with individual weight status, as simplistic interventions aimed at limited aspects of the physical environment may not provide the desired changes in obesity-related behaviours, let alone outcomes such as weight status. There is, for example, the potential for compensatory behaviours: more active people might consume more food, or people who use active transport may reduce physical activity in other domains of their lives. It needs to be understood which health-related activities people conduct where, when, for how long, with whom and so on, and also to include thorough appraisal of the different tools that measure perceptions of the environment in terms of validity, reliability and applicability.

### Strengths and limitations

Strengths of the present review are the adherence to essential criteria for literature reviews suggested by Ding and Gebel [12], the systematic assessment of methodological quality of the primary studies and the inclusion of articles that have been published since previous reviews. In addition, we stratified the results by continent and mode of measurement. However, there are also a number of limitations to this systematic literature review. We aimed to improve the methodological quality of the systematic literature review, but the quality assessment tool posed a number of challenges. First, it was difficult to assess the representativeness of the study samples. There is no consensus as to whether one should judge the representativeness on the response rate, on the sample size or on the characteristics of the sample. The assessment of the representativeness of the sample can be context-specific. Second, some papers did not present all necessary information, but instead referred to a design article published elsewhere. As the current review assessed the quality of the reviewed studies based on what was reported in the original publications - and it may be that not all relevant information regarding the quality criteria was reported - lower scores on the quality assessment may not necessarily reflect a low quality of the study but might merely have been a lack of reported detail in the paper. Third, the comparison between strong, moderate and weak articles in terms of finding results consistent with the hypothesis may have been hindered by publication or reporting bias.

Finally, by excluding articles that assessed physical environmental factors as potential mediators only, the selection of articles included in this review may be biased towards positive findings. Indeed, authors may respond to null or unexpected findings by changing the emphasis of their manuscript (for example using the physical environmental factor as confounder only). As a result, the inclusion of articles that treated physical environmental factors as confounders would be likely to strengthen our conclusion that the overall evidence for an association between environmental factors and weight status is weak.

## Conclusions

We systematically assessed the methodological quality of the included studies and took this quality into account in the review and interpretation of the evidence. The results of the present review remain in line with previous literature reviews [4,7,9,12], indicating that this additional step did not lead to different conclusions.

This systematic review provides an updated overview of the studies examining associations between the physical environment and weight status. We add to the existing literature by stratifying articles by continent and mode of measurement. The fact that this extensive review showed minimal evidence for an association between characteristics of the built environment and weight status indicates that we still do not fully understand the complex relations involved.

Although land use mix and urban sprawl were more consistently associated with overweight or obesity than other physical environmental factors, the evidence remains weak and the nature of associations between the physical environment and weight status needs further study.

## Abbreviations

BMI: Body mass index; EBRB: Energy balance related behaviour.

## Competing interests

The authors declare that they have no competing interest.

## Authors’ contributions

JDM carried out the literature search, performed the quality assessment of included articles and drafted the manuscript. HR and JL helped draft the manuscript. KG performed the quality assessment as second reviewer. All authors commented on and approved the final manuscript.

## Pre-publication history

The pre-publication history for this paper can be accessed here:

http://www.biomedcentral.com/1471-2458/14/233/prepub

## Supplementary Material

Additional file 1 Search strategy. Click here for file

Additional file 2 Quality Assessment Tool. Click here for file

Additional file 3 Flowchart. Click here for file

Additional file 4 Quality assessment of the included studies. Click here for file
