# Supplementary material for: Obesogenic environments: a systematic review of the association between the physical environment and adult weight status, the SPOTLIGHT project
Source: BMC Public Health. 2014 Mar 6;14:233. doi: 10.1186/1471-2458-14-233 (PMC4015813; doi:10.1186/1471-2458-14-233)
Supplement: Additional file 1 — Search strategy. [file 1471-2458-14-233-S1.docx]

Additional file 1

Search strategy Review Physical environmental determinants of obesity:

**Search terms**:

Obesity

Overweight

Weight status

Body Composition

Body Mass Index

Adiposity

Weight Loss

Waist Circumference

BMI

Environmental influence(s)

Environmental determinant(s)

Environmental factor(s)

Environmental support

Environmental approach

Environmental variable(s)

Environmental attribute(s)

Environmental barrier(s)

Environmental characteristic(s)

Environmental correlate(s)

Local environment

Rural environment

Urban environment

Objective environment

Perceived environment

Measured environment

Obesogenic environment

Residence characteristics

Environment design

Neighbo(u)rhoud characteristics

Geospatial

GIS

Food outlet

Geographic Information System(s)

Graffiti

Aesthetics

Streetlights

Walkability

Accessibility

Street connectivity

City planning

Built environment(s)

Park(s)

Sidewalk(s)

Green space

Sprawl

Land use mix

grocery store(s)

proximity

population density

retail density

fast food density

spatial access

recreational facilities

active transport

worksite

neighbo(u)rhood planning

pedestrian

urban design

In: healthy adults of the general population, published between 1995 and now, in English, French, German or Dutch.

Example of search in Pubmed:

#1: Obesity[Mesh] OR obesity[tiab] OR overweight[Mesh] OR overweight[tiab] OR “body composition”[Mesh] OR “fat distribution”[Mesh] OR “body mass index”[Mesh] OR adiposity[Mesh] OR “weight loss”[Mesh] OR “waist circumference”[Mesh] OR “body mass index”[tiab]

AND

#2: “environmental influence”[tiab] OR “environmental influences”[tiab] OR “environmental determinant”[tiab] OR “environmental determinants”[tiab] OR “environmental factor”[tiab] OR “environmental factors”[tiab] OR “environmental support”[tiab] OR “environmental approach”[tiab] OR “environmental variable”[tiab] OR “environmental variables”[tiab] OR “environmental attribute”[tiab] OR “environmental attributes”[tiab] OR “environmental barrier”[tiab] OR “environmental barriers”[tiab] OR “environmental characteristic”[tiab] OR “environmental characteristics”[tiab] OR OR “environmental correlates”[tiab] OR “local environment”[tiab] OR “rural environment”[tiab] OR “urban environment”[tiab] OR “objective environment”[tiab] OR “perceived environment”[tiab] OR “measured environment”[tiab] OR “obesogenic environment”[tiab] OR “residence characteristics”[tiab] OR “environment design”[tiab] OR “neighborhood characteristics”[tiab] OR “neighbourhood characteristics”[tiab] OR geospatial[tiab] OR GIS[tiab] OR “food outlet”[tiab] OR “geographic information system”[tiab] OR “geographic information systems”[tiab] OR graffiti[tiab] OR aesthetics[tiab] OR streetlights[tiab] OR walkability[tiab] OR “accessibility”[tiab] OR “street connectivity”[tiab] OR “Cities/epidemiology”[Mesh] OR “city planning”[tiab] OR “built environment”[tiab] OR “built environments”[tiab] OR “physical environment”[tiab] OR park[tiab] OR parks[tiab] OR sidewalk[tiab] OR “green space”[tiab] or sprawl[tiab] OR “land use”[tiab] OR “grocery store”[tiab] OR “grocery stores”[tiab] OR proximity[tiab] OR “population density”[tiab] OR “retail density”[tiab] OR “fast food density”[tiab] OR “spatial access”[tiab] OR “recreational facilities”[tiab] OR “active transport”[tiab] OR worksite[tiab] OR worksites[tiab] OR “sports facilities”[tiab] OR “urban form”[tiab] OR “zoning”[tiab] OR “neighbourhood planning”[tiab] OR “neighborhood planning”[tiab] OR pedestrian[tiab] OR “urban design”[tiab]

NOT

#3: ((child[Mesh] OR adolescent[Mesh] OR youth*[tiab] OR childhood[tiab] OR adolescent*[tiab] OR infant*[tiab]) NOT adult[Mesh])

AND

Filters activated: Publication date from 1995/01/01 to 2013/12/31, German, French, English, Dutch
