# Supplementary material for: Obesogenic environments: a systematic review of the association between the physical environment and adult weight status, the SPOTLIGHT project
Source: BMC Public Health. 2014 Mar 6;14:233. doi: 10.1186/1471-2458-14-233 (PMC4015813; doi:10.1186/1471-2458-14-233)
Supplement: Additional file 3 — Flowchart. [file 1471-2458-14-233-S3.docx]

Additional file 3

Flow chart of the literature search

Cochrane Library

624

EMBASE

5,261

PsycINFO

2

Web of Science

2,969

PubMed

3,839

Identification

12,844 articles

Duplicates removed

5,642 unique articles

Screening

Exclusion based on title and abstract

Reviewed

212

Eligibility

Exclusion based on full text:

- no weight status (6)
- only spatial or trend analysis (6)
- rural areas (13)
- one specific factor (2)

- no neighbourhood

physical environmental factors (13)

- no primary data analysis (24)
- only social environment (11)
- no individual data (5)
- no representative sample (14)
- # of participants too low (11)
- studies not conducted in high income countries (15)

Selected

92

Inclusion
