# Supplementary material for: Obesogenic environments: a systematic review of the association between the physical environment and adult weight status, the SPOTLIGHT project
Source: BMC Public Health. 2014 Mar 6;14:233. doi: 10.1186/1471-2458-14-233 (PMC4015813; doi:10.1186/1471-2458-14-233)
Supplement: Additional file 4 — Quality assessment of the included studies. [file 1471-2458-14-233-S4.docx]

Additional file 4

Quality assessment of the included studies

| **Authors** | **Year** | **Country** | **Study design*** | **Representa-tiveness I*** | **Representa-tiveness II*** | **Confounding*** | **Data collection*** | **Data analysis*** | **Reporting*** | **Overall rating*** |
| --- | --- | --- | --- | --- | --- | --- | --- | --- | --- | --- |
| Bai et al. | 2013 | USA | **2** | **1** |  | **1** | **2** | **1** | **1** | **1** |
| Ball et al. | 2012 | UK | **2** | **1** |  | **1** | **2** | **1** | **1** | **1** |
| Berry et al. | 2010 | USA | **2** | **2** | **1** | **1** | **1** | **1** | **1** | **1** |
| Berry et al. | 2010 | USA | **2** | **2** | **1** | **1** | **1** | **1** | **1** | **1** |
| Brown et al. | 2013 | USA | **2** |  |  | **1** | **1** | **1** | **1** | **1** |
| Catlin et al. | 2003 | USA | **2** | **1** |  | **1** | **2** | **1** | **1** | **1** |
| Drewnowski | 2012 | USA | **2** | **1** |  | **1** | **2** | **1** | **1** | **1** |
| Ewing et al. | 2003 | USA | **2** | **1** |  | **2** | **1** | **1** | **1** | **1** |
| Frank et al. | 2009 | USA | **2** | **1** |  | **1** | **2** | **1** | **1** | **1** |
| Gebel et al. | 2011 | Australia | **2** | **1** | **1** | **1** | **2** | **1** | **1** | **1** |
| Gibson | 2011 | USA | **2** | **1** | **2** | **1** | **2** | **1** | **1** | **1** |
| Giles-Corti et al. | 2003 | Australia | **2** | **1** |  | **1** | **1** | **1** | **1** | **1** |
| Hutchinson et al. | 2012 | USA | **2** | **1** |  | **1** | **2** | **1** | **1** | **1** |
| James et al. | 2012 | USA | **2** | **1** |  | **2** | **2** | **1** | **1** | **1** |
| Joshu et al. | 2007 | USA | **2** | **1** |  | **1** | **1** | **1** | **1** | **1** |
| Kestens et al. | 2012 | Canada | **2** | **1** |  | **1** | **1** | **1** | **1** | **1** |
| Leal et al. | 2011 | France | **2** | **1** |  | **1** | **2** | **1** | **1** | **1** |
| McDonald et al. | 2010 | USA | **2** | **2** |  | **1** | **1** | **1** | **1** | **1** |
| Mehta et al. | 2008 | USA | **2** | **1** |  | **1** | **2** | **1** | **1** | **1** |
| Mujahid et al. | 2008 | USA | **2** | **2** |  | **1** | **1** | **1** | **1** | **1** |
| Poortinga | 2006 | UK | **2** | **2** |  | **1** | **1** | **1** | **2** | **1** |
| Powell-Wiley et al. | 2013 | USA | **2** | **2** |  | **1** | **1** | **1** | **1** | **1** |
| Prince et al. | 2011 | Canada | **2** | **1** |  | **1** | **1** | **1** | **1** | **1** |
| Toftager et al. | 2011 | Denmark | **2** | **1** |  | **1** | **2** | **1** | **1** | **1** |
| Van Dyck et al. | 2010 | Belgium | **2** | **1** |  | **1** | **1** | **1** | **1** | **1** |
| West et al. | 2012 | USA | **2** | **2** |  | **1** | **1** | **1** | **1** | **1** |
| Wilson et al. | 2007 | USA | **2** | **1** |  | **1** | **1** | **1** | **1** | **1** |
| Yamada et al. | 2012 | USA | **2** | **1** |  | **1** | **2** | **1** | **1** | **1** |
| Zick et al.. | 2013 | USA | **2** | **1** |  | **1** | **2** | **1** | **1** | **1** |
| Ahern et al. | 2011 | USA | **2** | **3** |  | **1** | **2** | **1** | **2** | **2** |
| Black et al. | 2009 | USA | **2** | **1** |  | **1** | **3** | **1** | **1** | **2** |
| Black et al. | 2010 | USA | **2** | **2** |  | **1** | **2** | **1** | **1** | **2** |
| Block et al. | 2011 | USA | **2** | **2** | **2** | **1** | **3** | **1** | **1** | **2** |
| Boehmer et al. | 2007 | USA | **2** | **1** | **2** | **2** | **2** | **1** | **1** | **2** |
| Brown et al. | 2009 | USA | **2** | **1** |  | **1** | **3** | **1** | **1** | **2** |
| Casagrande et al. | 2011 | USA | **2** | **2** | **3** | **2** | **2** | **1** | **1** | **2** |
| Chen et al. | 2012 | USA | **2** | **2** |  | **1** | **2** | **1** | **2** | **2** |
| Coombes et al. | 2010 | UK | **2** | **2** |  | **1** | **2** | **1** | **1** | **2** |
| Cummins & Fagg | 2012 | UK | **2** | **2** |  | **1** | **3** | **1** | **1** | **2** |
| Doyle et al. | 2006 | USA | **2** | **3** |  | **1** | **1** | **1** | **1** | **2** |
| Ellaway et al. | 2005 | Europe | **2** | **2** |  | **1** | **3** | **1** | **1** | **2** |
| Frank et al. | 2004 | USA | **2** | **2** |  | **1** | **3** | **1** | **1** | **2** |
| Frank et al. | 2008 | USA | **2** | **2** |  | **1** | **3** | **1** | **1** | **2** |
| Frank et al. | 2007 | USA | **2** | **2** |  | **1** | **3** | **1** | **1** | **2** |
| Frank et al. | 2007 | USA | **2** | **2** |  | **1** | **2** | **1** | **1** | **2** |
| Garden et al. | 2008 | Australia | **2** | **1** |  | **1** | **3** | **1** | **1** | **2** |
| Gregson | 2011 | USA | **2** | **2** |  | **1** | **3** | **1** | **2** | **2** |
| Hattori et al. | 2013 | USA | **2** | **1** |  | **1** | **3** | **1** | **1** | **2** |
| Hoehner et al. | 2011 | USA | **2** | **3** |  | **1** | **1** | **1** | **2** | **2** |
| Inagami et al. | 2009 | USA | **2** | **1** |  | **1** | **3** | **1** | **1** | **2** |
| Jeffery et al. | 2006 | USA | **2** | **1** |  | **1** | **3** | **1** | **1** | **2** |
| Keegan et al. | 2011 | USA | **2** | **1** | **3** | **1** | **2** | **1** | **1** | **2** |
| Lesser et al. | 2013 | USA | **2** | **2** |  | **1** | **3** | **1** | **1** | **2** |
| Lopez et al. | 2004 | USA | **2** | **2** |  | **1** | **3** | **1** | **1** | **2** |
| Lopez | 2007 | USA | **2** | **2** |  | **1** | **3** | **1** | **1** | **2** |
| Lovasi et al. | 2012 | USA | **2** | **2** |  | **1** | **3** | **1** | **1** | **2** |
| Lovasi et al. | 2009 | USA | **2** | **2** |  | **1** | **3** | **1** | **1** | **2** |
| MacDonald et al. | 2011 | UK | **2** | **2** |  | **1** | **3** | **1** | **1** | **2** |
| Mobley et al. | 2006 | USA | **2** | **3** |  | **1** | **3** | **1** | **1** | **2** |
| Morland et al. | 2006 | USA | **2** | **1** |  | **1** | **3** | **1** | **1** | **2** |
| Nielsen & Hansen | 2007 | Denmark | **2** | **1** |  | **1** | **3** | **1** | **1** | **2** |
| Oka et al. | 2012 | USA | **2** | **3** |  | **1** | **2** | **1** | **1** | **2** |
| Pearce et al. | 2009 | New Zealand | **2** | **1** |  | **1** | **3** | **1** | **1** | **2** |
| Plantinga & Bernell | 2007 | USA | **2** | **1** |  | **1** | **3** | **1** | **1** | **2** |
| Plantinga & Bernell | 2007 | USA | **2** | **1** |  | **1** | **3** | **1** | **1** | **2** |
| Pouliou and Elliott | 2010 | Canada | **2** | **1** |  | **1** | **3** | **1** | **1** | **2** |
| Prince et al. | 2012 | Canada | **2** | **1** |  | **1** | **3** | **1** | **1** | **2** |
| Richardson et al. | 2013 | New Zealand | **2** |  |  | **1** | **2** | **1** | **1** | **2** |
| Ross et al. | 2007 | Canada | **2** | **2** |  | **1** | **2** | **1** | **1** | **2** |
| Rundle et al. | 2007 | USA | **2** | **2** |  | **1** | **1** | **1** | **1** | **2** |
| Rundle et al. | 2008 | USA | **2** | **1** |  | **1** | **3** | **1** | **1** | **2** |
| Rundle et al. | 2008 | USA | **2** | **2** |  | **1** | **3** | **1** | **1** | **2** |
| Rundle et al. | 2013 | USA | **2** |  |  | **1** | **3** | **1** | **1** | **2** |
| Rutt et al. | 2004 | USA | **2** | **3** |  | **1** | **1** | **1** | **1** | **2** |
| Sallis et al. | 2009 | USA | **2** | **1** |  | **1** | **3** | **1** | **1** | **2** |
| Santana et al. | 2009 | Portugal | **2** | **2** |  | **1** | **2** | **1** | **1** | **2** |
| Scott et al. | 2009 | USA | **2** | **1** |  | **1** | **3** | **1** | **1** | **2** |
| Smith et al. | 2008 | USA | **2** | **1** |  | **1** | **3** | **1** | **1** | **2** |
| Smith et al. | 2011 | USA | **2** | **1** |  | **1** | **3** | **1** | **1** | **2** |
| Spence et al. | 2009 | Canada | **2** | **1** |  | **1** | **3** | **1** | **1** | **2** |
| Tilt et al. | 2007 | USA | **2** | **1** |  | **1** | **3** | **1** | **1** | **2** |
| Wang et al. | 2007 | USA | **2** | **1** |  | **1** | **3** | **1** | **1** | **2** |
| Zick et al. | 2009 | USA | **2** |  |  | **1** | **3** | **1** | **1** | **2** |
| Bodea et al. | 2008 | USA | **2** | **2** | **3** | **1** | **3** | **1** | **1** | **3** |
| Bodor et al . | 2010 | USA | **2** | **1** | **3** | **3** | **3** | **1** | **1** | **3** |
| Burgoine et al. | 2011 | UK | **2** | **2** | **3** | **2** | **3** | **1** | **1** | **3** |
| Christian et al. | 2011 | Australia | **2** | **3** |  | **1** | **3** | **1** | **1** | **3** |
| Eid et al. | 2008 | USA | **2** | **3** | **2** | **1** | **3** | **1** | **2** | **3** |
| Jilcott et al. | 2010 | USA | **2** | **3** |  | **1** | **3** | **2** | **2** | **3** |
| Samimi et al. | 2009 | USA | **2** | **3** |  | **1** | **2** | **1** | **1** | **3** |
| Zhao and Kaestner | 2010 | USA | **2** | **3** | **3** | **1** | **3** | **1** | **1** | **3** |
| Pendola and Gen | 2007 | USA | **2** | **1** |  | **1** | **3** | **2** |  |  |

**NB.** Scores for risk-of-bias range from 1 (low risk of bias; high methodological quality) to 3 (high risk of bias; low methodological quality). Articles that are shaded grey were those that only reported outcomes in agreement with the hypothesis.

* Study design refers to the distinction between experimental and observational, and cross-sectional and longitudinal studies. Representativeness I refers to the extent to which the study population is generalizable to the population it was drawn from. Representativeness II refers to representativeness due to withdrawal and dropout in longitudinal designs. Confounding refers to the adjustment for relevant confounding factors in analyses. Data collection refers to the extent in which valid and reliable instruments were used for data collection. Data analysis refers to the methods used for data analysis (for example only descriptive analyses versus more complex analyses). Data reporting refers to the extent in which authors were specific in their reporting about hypotheses and probability values.
